# Cellular cholesterol delivery, intracellular processing and utilization for biosynthesis of steroid hormones

**DOI:** 10.1186/1743-7075-7-47

**Published:** 2010-06-01

**Authors:** Jie Hu, Zhonghua Zhang, Wen-Jun Shen, Salman Azhar

**Affiliations:** 1 Geriatric Research, Education and Clinical Center, VA Palo Alto Health Care System, Palo Alto, California 94304, USA; 2 Stanford University, Stanford, California 94305, USA; 3 Norris Cotton Cancer Center, Dartmouth Medical School, 1 Medical Center Drive, Lebanon, NH 03756, USA

## Abstract

Steroid hormones regulate diverse physiological functions such as reproduction, blood salt balance, maintenance of secondary sexual characteristics, response to stress, neuronal function and various metabolic processes. They are synthesized from cholesterol mainly in the adrenal gland and gonads in response to tissue-specific tropic hormones. These steroidogenic tissues are unique in that they require cholesterol not only for membrane biogenesis, maintenance of membrane fluidity and cell signaling, but also as the starting material for the biosynthesis of steroid hormones. It is not surprising, then, that cells of steroidogenic tissues have evolved with multiple pathways to assure the constant supply of cholesterol needed to maintain optimum steroid synthesis. The cholesterol utilized for steroidogenesis is derived from a combination of sources: 1) *de novo *synthesis in the endoplasmic reticulum (ER); 2) the mobilization of cholesteryl esters (CEs) stored in lipid droplets through cholesteryl ester hydrolase; 3) plasma lipoprotein-derived CEs obtained by either LDL receptor-mediated endocytic and/or SR-BI-mediated selective uptake; and 4) in some cultured cell systems from plasma membrane-associated free cholesterol. Here, we focus on recent insights into the molecules and cellular processes that mediate the uptake of plasma lipoprotein-derived cholesterol, events connected with the intracellular cholesterol processing and the role of crucial proteins that mediate cholesterol transport to mitochondria for its utilization for steroid hormone production. In particular, we discuss the structure and function of SR-BI, the importance of the selective cholesterol transport pathway in providing cholesterol substrate for steroid biosynthesis and the role of two key proteins, StAR and PBR/TSO in facilitating cholesterol delivery to inner mitochondrial membrane sites, where P450scc (CYP11A) is localized and where the conversion of cholesterol to pregnenolone (the common steroid precursor) takes place.

## Introduction

**Figure 1:**
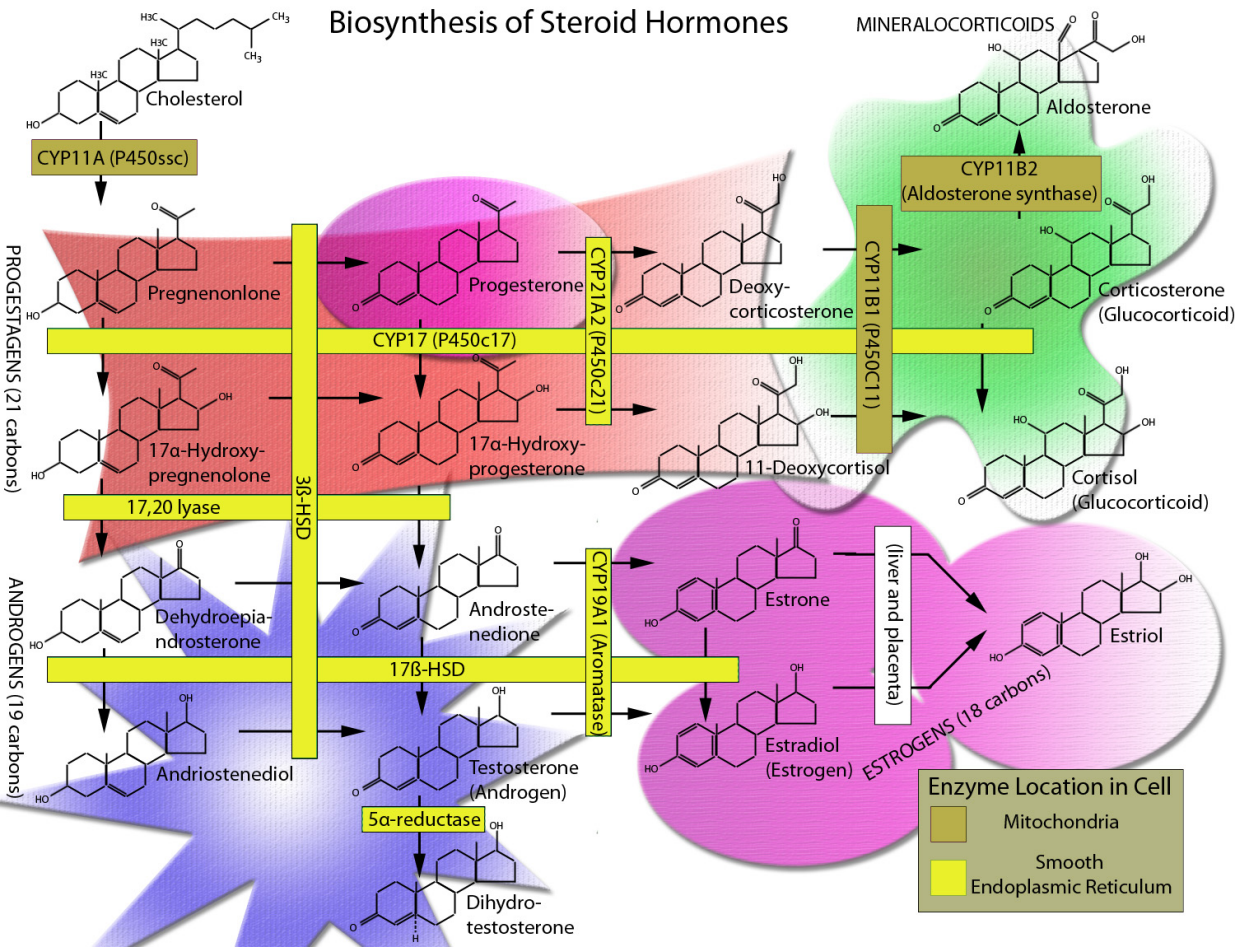
**Principal steps involved in the biosynthesis of various steroid hormones**. Modified from Payne and Hales and website [1,305]

Cholesterol is a starting material for the biosynthesis of steroid hormones; these fat soluble, low molecular weight substances play diverse and important physiological functions (Table 1). There are five major classes of steroid hormones: testosterone (androgen), estradiol (estrogen), progesterone (progestin), cortisol/corticosterone (glucocorticoid), and aldosterone (mineralocorticoids). Testosterone and its more potent metabolite dihydrotestosterone (DHT), progesterone and estradiol are classified as sex-steroids, whereas cortisol/corticosterone and aldosterone are collectively referred to as corticosteroids [1-3]. All these steroid hormones are synthesized from cholesterol through a common precursor steroid, pregnenolone [1-3], which is formed by the enzymatic cleavage of a 6-carbon side-chain of the 27-carbon cholesterol molecule, a reaction catalyzed by the cytochrome P450 side-chain cleavage enzyme (P450scc, CYP11A1) (Fig. 1) [4-6]. The adrenal gland produces both corticosteroids and androgens (dihydroepiandosterone [DHEA], and androstenedione); aldosterone is mainly produced by the cells of the zona glomerulosa layer, cortisol/corticosterone is principally produced by the adrenocortical cells of the zona fasciculata layer and adrenal DHEA whereas androstenedione is synthesized by cells of the zona reticularis layer (Table 1) [1,7-9]. The ovarian granulosa cells mainly secrete progesterone (and its metabolite 20**a**-hydroxyprogesterone) and estradiol; ovarian theca cells predominantly synthesize androgens, and ovarian luteal cells secrete progesterone (and its metabolite 20α-hydroxyprogesterone), while testicular Leydig cells are the site of testosterone production (Table 1) [1,7-9]. Progesterone is also synthesized by the corpus luteum during the first 6-8 weeks of gestation, but during pregnancy the main source of progesterone is the placenta [10,11]. The brain also synthesizes steroids *de novo *from cholesterol through mechanisms that are at least partly independent of peripheral steroidogenic cells [[12-14] and references there in]. Such *de novo *synthesized brain steroids are commonly referred to as neurosteroids [12-14].

**Table 1:** Major steroids and their physiological functions

Steroidogenic Tissues	Trophic Hormone	Steroids(s)	Physiological Functions
**Ovary**			
Granulosa cells	FSH	Estradiol	Estrogen, a principal female sex steroid, required for growth and ovulation, responsible for secondary female sex characteristics, regulator of cardiovascular physiology, bone integrity and neuronal growth
Luteinized Granulosa/luteal Cells	LH	Progesterone	A progestin, required for follicular growth and ovulation, responsible for changes associated with luteal phase of the menstrual cycle, essential for the establishment and maintenance of early pregnancy
Theca-interstitial Cells	LH	Testosterone Androstenedione	Androgens, precursors for estrogens, transported into granulosa cells, where they are converted into estardiol and other estrogens by aromatase (CYP19A1) enzyme
**Testis**			
Leydig cells	LH	Testosterone	The most prevalent male sex hormone (androgen); testosterone and its biologically active form, dihydrotestosterone (DHT) are necessary for normal spermatogenesis and development, responsible for secondary sex characteristics, responsible for increased muscle mass, sexual function, body hair and decreased risk of osteoporosis
**Adrenal gland**			
Z. glomerulosa Cells	ACTH, K^+ ^Angiotensin II	Aldosterone	The principal mineralocorticoid, raises blood pressure and fluid volume, enhances sodium reabsorption in the kidney, sweat gland, stomach and salivary gland and also enhances excretion of potassium and hydrogen ions from the kidney.
Z. glomerulosa Cells	ACTH	Cortisol	The dominant glucocorticoid in humans (in rodents, the major glucocorticoid is corticosterone), elevates blood pressure and Na^+ ^uptake, involved in stress adaptation, regulates carbohydrate, protein and lipid metabolism nearly opposite to that of insulin, influences inflammatory reactions and numerous effects on the immune system.
Z. reticularis Cells	ACTH POC-derived peptide Other factors	Androstenedione DHEA DHEA-sulfate	The function of adrenal androgens is not well understood, except that they contribute to the maintenance of secondary sex characteristics, may also be involved in the regulation of bone mineral density, muscle mass and may beneficial actions against type 2 diabetes and obesity
**Placenta**	Peptide growth Factors, cAMP	Progesterone Estrogens	Maintenance of pregnancy
**Brain**			
Neurons, Glial cells Purkinje cells	Neurotransmitters Neuropeptides	Progesterone Estradiol, DHEA, ALLO, THDOC	Neurosteroids are implicated in various processes such as proliferation, differentiation, activity and survival of nerve cells and a variety of neuronal functions including control and behavior, neuroendocrine and metabolic processes.

**Figure 2:**
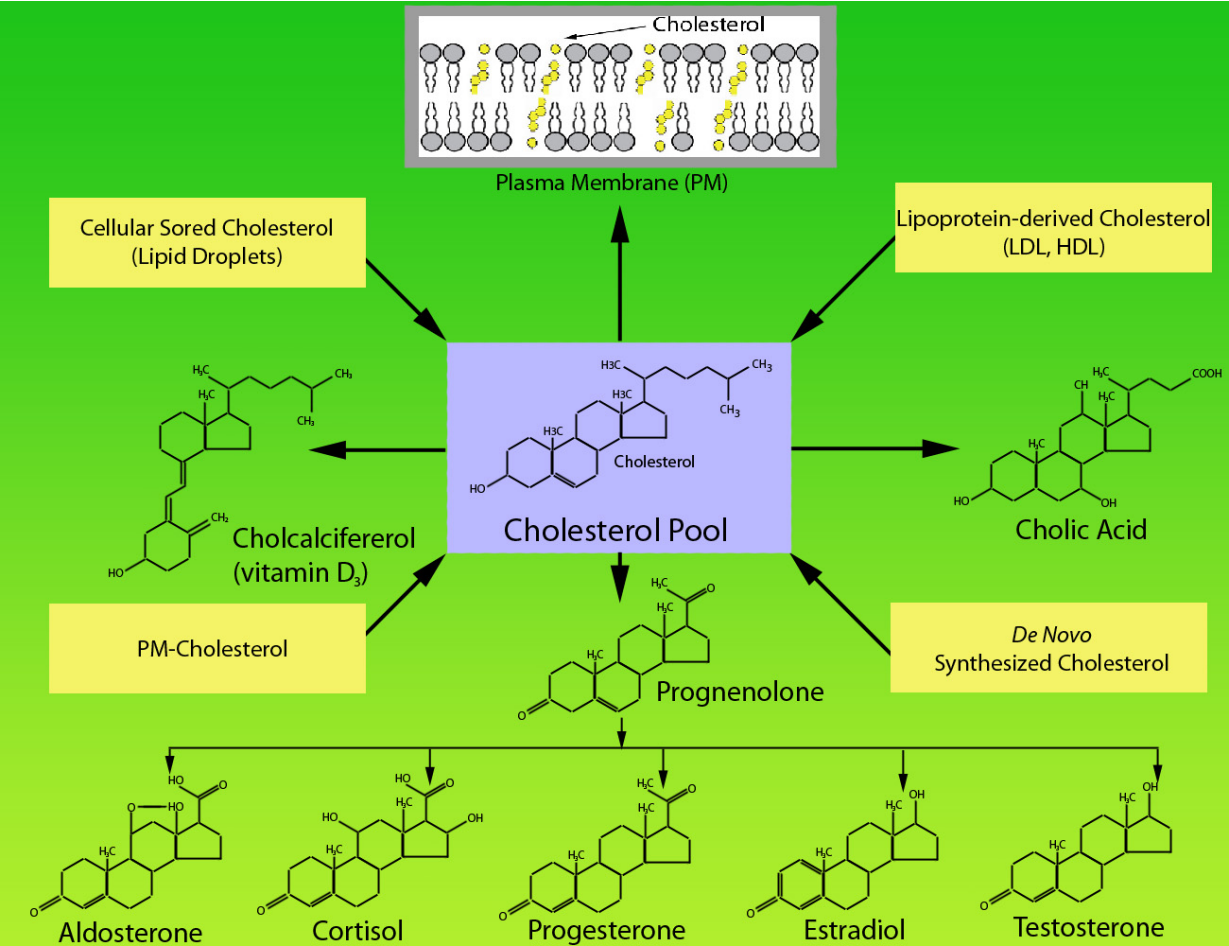
Potential sources of cholesterol for product formation (steroids, vitamin D and bile acids) and membrane biogenesis

Although adrenal, ovarian and testicular steroidogenesis is primarily under the control of tissue-specific tropic hormones (discussed below); the availability of adequate cholesterol substrate is also a critical requirement for the optimal steroid hormone production. The steroidogenic tissues and cells have the potential to obtain cholesterol for steroid synthesis from at least four potential sources (Fig. 2): *a*) cholesterol synthesized *de novo *from acetate; *b*) cholesterol obtained from plasma low-density lipoprotein (LDL) and high-density lipoprotein (HDL); *c*) cholesterol-derived from the hydrolysis of stored cholesterol esters in the form of lipid droplets; and d) cholesterol interiorized from the plasma membrane. Although all three major steroidogenic organs (adrenal, testis and ovary) can synthesize cholesterol *de novo *under the influence of the tropic hormone, the adrenal and ovary preferentially utilize cholesterol supplied from plasma LDL and HDL via the LDL-receptor mediated *endocytic *pathway and SR-BI-mediated *selective *pathway, respectively [9,15-20]. The use of LDL or HDL as the source of cholesterol for steroidogenesis appears to be species dependent; rodents preferentially utilize the SR-BI/selective pathway while humans, pigs and cattle primarily employ the LDL/LDL-receptor endocytic pathway to meet their cholesterol need for steroid synthesis. In contrast, testicular Leydig cells under normal physiological conditions rely heavily on the use of endogenously synthesized cholesterol for androgen (testosterone) biosynthesis [9,20].

This review is focused on the role of cholesterol in the regulation of steroidogenesis. We first present an overview of various enzymatic pathways involved in the conversion of cholesterol to tissue-specific steroid hormones. Next, we summarize our current understanding about the molecules and processes that participate in the uptake of plasma lipoprotein-derived cholesterol with particular emphasis on the SR-BI/selective cholesterol transport pathway, events connected with the intracellular processing and trafficking of cholesterol and key proteins which facilitate the transport of cholesterol to and within the mitochondria for steroid synthesis.

## Biosynthesis of steroid hormones--an overview

The overall rate of steroidogenesis (i.e., steroid hormone production) is controlled by tropic (peptide) hormones [21-26]. The type of steroid hormone that can be synthesized by a particular cell type is dictated by its complement of peptide hormone receptor, its response to peptide hormone stimulation and its genetically expressed complement of steroiodgenic enzymes (Fig. 1). Thus, adrenocorticotropic hormone (ACTH) stimulates cortisol/corticosterone in adrenocortical fasciculata-reticularis cells, angiotensin II (AII) and potassium regulate aldosterone synthesis in adrenal glomerulosa cells, follicle-stimulating hormone (FSH) controls the progesterone and estrogen synthesis in ovarian granulosa cells, whereas luteinizing hormone (LH) regulates progesterone synthesis in luteinized ovarian granulosa-luteal cells, androgen production in ovarian theca-interstitial cells and testosterone synthesis in testicular Leydig cells (Table 1) [27-38]. The adrenal gland is also responsible for the synthesis of adrenal androgens [39,40]. Tropic hormones (LH, FSH or ACTH) induce adrenocortical and gonadal steroidogenesis by binding to their respective G protein-coupled receptors, leading to activation of adenylate cyclase, which generates cAMP and activates cAMP-dependent protein kinase (PKA) [21-25]. Stimulation of the cAMP-PKA signaling cascade exerts both acute and chronic effects on the regulation of steroid hormone production. The acute steroidogenic response, which occurs on the order of minutes, is characterized by a rapid mobilization of lipid droplet stored CEs and increased delivery of cholesterol to the mitochondrial cytochrome P450 cholesterol side-chain cleavage (P450scc) enzyme (encoded by CYP11A1) followed by rapid synthesis of new steroids. More chronic, long-term regulation of steroidogenesis also occurs at the level of the transcription of the genes for the steroidogenic enzymes to enhance, which results in the enhanced synthetic capacity of the cell [41-45]. Note: angiotensin (AII) stimulation of aldosterone biosynthesis in adrenal glomerulosa cells is primarily mediated by the protein kinase C signaling cascade, whereas potassium stimulation of aldosterone production also involves Ca^2+^-calmodulin-dependent kinase [26].

Although the final steroid product differs for these several cell types (described above), the first committed reaction in the biosynthetic pathway is the same, i.e., the conversion of cholesterol to pregnenolone by the cytochrome P450 cholesterol side-chain cleavage (P450scc) enzyme (CYP11A1). P450scc is an enzyme complex consisting of a flavoprotein (NADH-adrenodoxin reductase), a ferredox (adrenodoxin) and a cytochrome P450 localized on an inner mitochondrial membrane [3,7]. P450scc catalyzes three distinct reactions: 20α-hydroxylation, 22-hydroxylation and scission of 20, 22 carbon-carbon bond, thus converting cholesterol to pregnenolone [3,7]. This initial step in steroid hormone (pregnenolone) synthesis also represents a rate limiting step. The rate limiting nature of this step does not result from a limitation of the P450scc activity itself (conversion of cholesterol to pregnenolone) but from limitation of access of cholesterol to the substrate site of P450scc, i.e., delivery of substrate cholesterol from an outer to an inner mitochondrial membrane where P450scc resides [46-50].

The pregnenolone produced in the rate-limiting step is further exposed to endoplasmic reticulum and mitochondria for further modifications [1,2,7,51]. It is first converted to progesterone by the enzyme Δ^5^-3β hydroxysteroid dehydrogenase isomerase (3βHSD), which is also one of the main steroids produced by the steroidogenic cells of the ovary. In zona fasciculata cells of the adrenal cortex, progesterone is hydroxylated to 17α-hydroxyprogesterone by P450c17 (CYP17), which is subsequently metabolized to 11-deoxycortisol (or deoxycorticosterone) by P450c21 (CYP21A2). The final step in cortisol biosynthesis takes place in the mitochondria and involves the conversion of 11-deoxcortisol (deoxycorticosterone) to cortisol or to corticosterone in rodents by the enzyme P450c11 (CYP11B1). The next two steps in aldosterone biosynthesis are catalyzed by aldosterone synthase (CYP11B2), which converts 11-deoxycorticosterone to corticosterone and subsequently to aldosterone. In testicular Leydig cells, pregnenolone is converted to testosterone via two pathways known as the Δ^4 ^and Δ^5 ^pathways. The relative activities of the two pathways are known to vary according to species [1]. The Δ^4 ^involves sequential conversion of pregnenolone to progesterone to 17α-hydroxyprogesterone to androstenedione to testosterone, while in the Δ^5 ^pathway pregnenolone is converted to 17α-hydroxypregnenolone to dehydroepiandrosterone to testosterone through either androstenediol or androstenedione. In the ovary, testosterone is further metabolized to estradiol, a reaction catalyzed by aromatase (CYP19A1). In addition, estradiol can also be formed through combined actions of aromatase (CYP19A1) and 17-hydroxysteroid dehydrogenase (17HSD1) (Fig. 1).

## *De novo *cholesterol synthesis

Like many tissues, all steroid producing tissues and cells are capable of synthesizing cholesterol *de novo *[9,15-18,20]. Biosynthesis of 27-carbon skeleton of cholesterol involves the conversion of acetate (acetyl CoA) through a series of complex enzymatic steps requiring the participation of numerous enzymes [52]. Among the major steps, mevalonate is formed by the condensation of 3 molecules of acetyl-CoA, a reaction catalyzed by the rate limiting enzyme HMG-CoA-reductase, which is converted to squalene, a 30-carbon linear structure followed by cyclization to yield lanosterol and subsequently removal of 3 carbons to produce cholesterol. The endoplasmic reticulum (ER)-associated integral membrane protein complex, SCAP/SREBP, transcriptionally controls the expression of the genes of many enzymes involved in cholesterol biosynthesis including the rate-limiting enzyme, HMG-CoA reductase [52,53]. In steroidogenic cells, the *de novo *cholesterol biosynthesis is also under the control of tropic hormone [15-18]. Indeed, adrenal, ovarian and testicular Leydig cell cholesterol biosynthesis as well as HMG-CoA reductase is rapidly stimulated upon exposure to tropic hormone [15-18]. Newly synthesized cholesterol primarily moves initially from the ER to the plasma membrane (PM) [54-57]. This energy dependent and predominantly nonvesicular trafficking process appears to require the participation of cholesterol-rich, sphingolipid-rich domains (i.e., lipid rafts/caveolae) and proteins such as caveolin, heat shock proteins and possibly other soluble sterol carrier candidate proteins such as OSBP, ORPs, SCP2, START domain containing proteins and phosphoinositides but not NCP1 [58-66]. After endogenously synthesized cholesterol is transported to the PM, its immediate fate is not well understood at present. While newly synthesized cholesterol is preferentially translocated to the PM, excess cellular cholesterol from other cellular organelles including PM is transported back to ER for esterification. The PM reverse cholesterol transport to ER is suggested to involve at least two pathways: a) a vesicular route via an endosome and/or Golgi; and b) a nonvesicular alternative route [55,66]. It should also be mentioned that retrograde transport of cellular cholesterol to ER and translocation of newly synthesized ER cholesterol to PM follow different itineraries [55,66]. Cholesterol esterification is primarily catalyzed by ER-localized ACAT1 and newly formed CEs are stored along with triglycerides in the core of cytoplasmic lipid droplets [66-68]. Little is known about the mechanisms that control the biogenesis of lipid droplets, but it is clear that they are synthesized at and bud off from ER [69-71]. In steroidogenic cells of adrenal, ovary and testis, both the formation and depletion of lipid droplets is hormonally regulated. As noted before, the lipid droplet-associated cholesterol serves as a source of substrate for steroid hormone synthesis in response to acute hormonal stimulation [72-81].

## Receptor-mediated uptake and internalization of plasma lipoprotein-derived cholesterol

### (a) LDL(B/E)-receptor-mediated endocytic uptake of LDL-cholesterol

**Figure 3:**
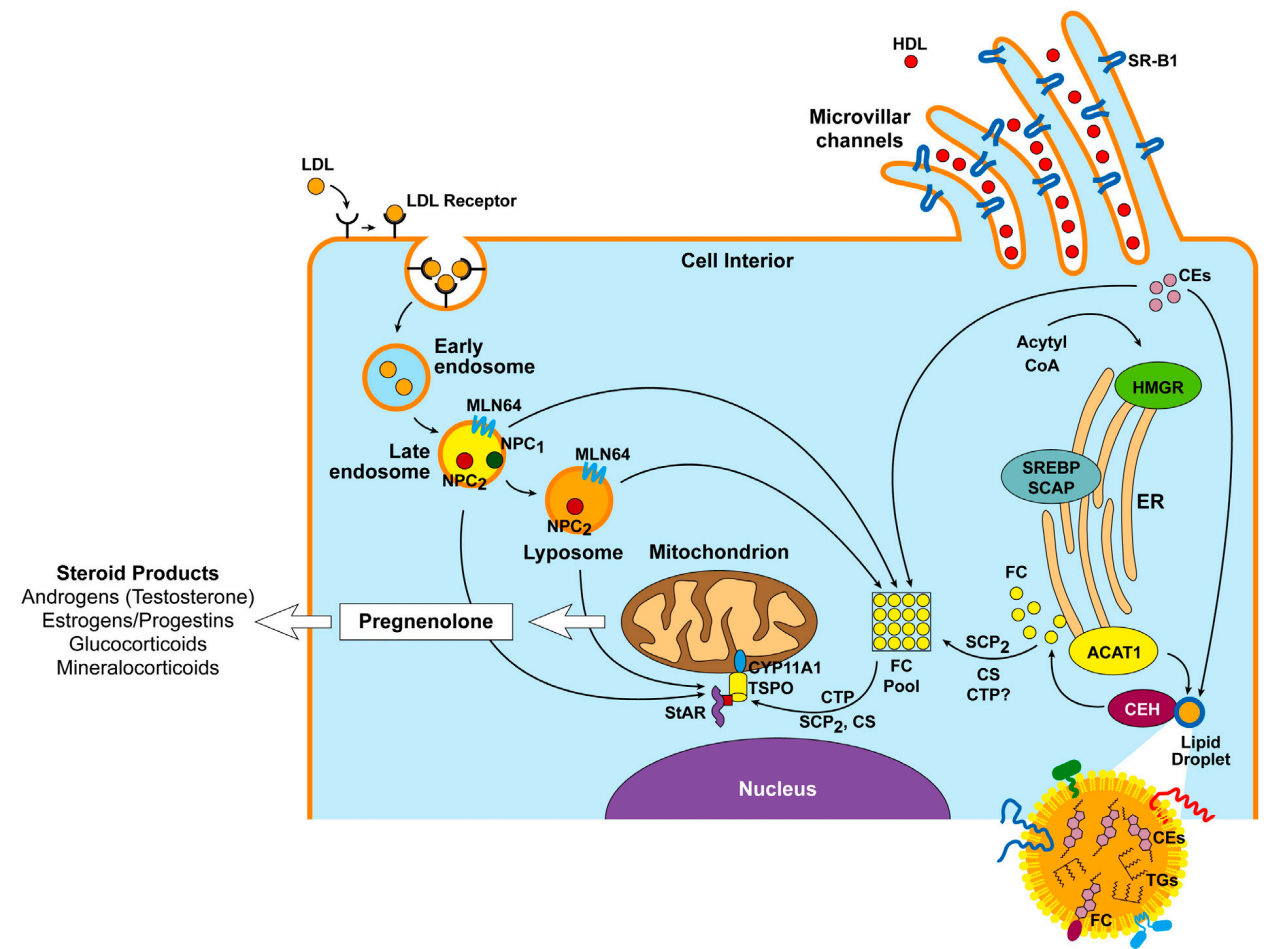
**Diagrammatic representation of the molecular and cellular events involved in the selective and endocytic uptake and intracellular processing of the lipoprotein-derived cholesteryl esters for steroid hormone biosynthesis by adrenal and gonadal tissues**. ACATA1, acyl-coenzyme A:cholesterol acyltransferase I; CEs, cholesteryl esters; CS, cytoskeleton; CYP11A1, cytochrome P450 side-chain cleavage enzyme (P450scc); FC, free cholesterol; NPC1, Nieman-Pick type C1; NPC2, Nieman-Pick type C2; SCP2, sterol carrier protein2; SREBP, sterol-regulatory element-binding proteins; SCAP, SREBP cleavage-activating protein. StAR, steroidogenic acute regulatory protein; TGs, triglycerides; TSPO, translocator protein. Modified from Chang *et al*, Rone *et al*, and Farese and Walther [68,288,306].

Although cellular *de novo *cholesterol synthesis and cholesteryl esters stored in lipid droplets can potentially supply adequate amounts of cholesterol substrate to support steroidogenesis, the overwhelming evidence now suggests that the adrenal and ovary (and testicular Leydig cells under certain conditions) preferentially utilize plasma lipoprotein-derived cholesterol for steroid synthesis [9,20]. One pathway by which steroid producing cells acquire cholesterol is from plasma LDL, or other apolipoprotein B- (apoB) or apoE-containing lipoproteins via the LDL (B/E) receptor-mediated endocytic pathway (Fig. 3) [19]. LDL or other relevant apoB/apoE lipoproteins bind to the LDL receptor initially localized at the plasma membrane (PM), which subsequently translocate to specialized regions of the plasma membrane, called the coated pits. The coated pits, made of clathrin protein, and some other accessory, invaginate, and pinch off from the PM in the form of coated vesicles [19,66]. These coated vesicles in the cell interior fuse with early endosomes, shed off their clathrin coat, and fuse with each other to form larger vesicles, called endosomes. At this stage, the LDL-receptor complex rapidly dissociates as the endosomal pH falls, and the released, but fully intact LDL-receptors are delivered to the endocytic recycling compartments (ERC) for their return itinerary to the PM [66]. The LDL cholesteryl esters (CEs) are hydrolyzed in a unique acid lipase-enriched compartment of early endosomes, the released cholesterol accumulates in the late endosomes/lysosomes, and subsequently through some unknown mechanisms is transported to PM as a membrane constituent, ER for esterification by the resident ER enzyme acyl-coenzyme A:cholesterol acyltransferase I (ACAT1) and storage in lipid droplets, or mitochondria for the synthesis of steroid hormones [67-71,82-85].

A pair of proteins called Nieman-Pick type C1 and C2 (NPC1 and NPC2, respectively) appear to be important in the movement of unesterified cholesterol out of the late endosomes and lysomes [66,86-89]. NPC1 is a polytopic, sterol-sensing protein of 1,278 amino acids located in the membranes of late endosomes and lysosomes that, along with NPC2, a cholesterol binding soluble protein of 131 amino acids located within lysosomes, facilitates the movement of cholesterol to various organelles by mechanisms not yet understood [66,88,89]. NPC1 appears to be important in trafficking LDL-cholesterol, particularly under conditions in which the substrate for steroidogenesis is primarily supported by LDL-cholesterol, but does not appear to be involved in other pathways for cholesterol delivery for steroidogenesis [90,91]. Additional proteins are also involved in this process such as MLN64 which facilitates the movement of lysosomal cholesterol to mitochondria for steroidogenesis [92,93]. MLN64 (StarD3) is a polytopic protein that is also found localized to late endosomes along with NPC1 and is a member of the StAR-related lipid transfer (START) domain superfamily that possesses cholesterol binding and transport activity [94]. However, targeted mutation of MLN64 in mice caused only minor alterations in sterol metabolism in vivo, but defects in cholesterol utilization for steroidogenesis were still seen *in vitro*, consistent with the existence of multiple mechanisms for cholesterol delivery for steroidogenesis. In addition, considerable evidence now indicates that transport of late endosomal/lysosomal LDL-cholesterol to other cellular destinations including mitochondria may also involve an endosomal-specific Rab 9 GTPase-dependent vesicular trafficking mechanism [66,95-97].

### (b) SR-BI-mediated 'selective' uptake of HDL-cholesterol

#### (i) Molecular characteristics and the functional expression of SR-BI

Whereas LDL-receptor-mediated uptake of cholesterol allows for its efficient delivery, steroidogenic cells can process exceptionally large quantities of lipoprotein-derived cholesteryl esters through a specialized pathway known as the "selective" cholesteryl ester uptake pathway (Fig. 3) [98-100]. In fact, the "selective" cholesterol uptake pathway is quantitatively the most important source for cholesterol delivery for steroidogenesis in the tropic-hormone stimulated rodent adrenal and ovary [9,20,99-102]. The term "selective" cholesterol uptake is used when cell surface bound cholesterol-rich lipoproteins (HDL or LDL, regardless of lipoprotein composition) release cholesteryl esters without the parallel uptake and lysosomal degradation of the lipoprotein particle itself [72,73,98-100,103]. Tropic hormone (ACTH or LH)-stimulated rodent adrenal and ovary (and under certain conditions testicular Leydig cells) rely heavily on selective cholesteryl ester uptake to fulfill their cholesterol needs for steroid synthesis [9,20,72,73,99-102]. The pathway is also functional in humans, in rodent liver and a variety of cultured cells such as isolated primary hepatocytes and hepatic cell lines, fibroblasts, adipocytes, and macrophages, although it may be quantitatively less important in humans [9,20].

Scavenger receptor class B, type I (SR-BI) is a physiologically relevant cell surface receptor responsible for "selective" uptake of lipoprotein-derived cholesteryl esters [104]. SR-BI is a member of the class B scavenger receptor family that also includes CD36, LIMPII, and SR-BII (an isoform of SR-BI with an alternate C-terminal cytoplasmic tail) [9,20,105]. SR-BI, like the other family members, contains two transmembrane domains, two cytoplasmic domains (the amino- and carboxyl terminal domains), as well as a large extracellular domain (ECD) containing a cysteine-rich region and multiple sites for N-linked glycosylation [9,20,105]. Cells, which have high levels of SR-BI, efficiently utilize the selective pathway in delivering cholesteryl esters for use in steroid hormones or product synthesis [9,20]. In rodents, SR-BI is abundantly expressed in the liver, but also in steroidogenic cells of the adrenal gland, ovary, and testis where SR-BI levels are regulated by tropic hormones and influence the selective uptake of HDL-CE, and ultimately, steroidogenesis in these organs [74,75,106-110].

It is also of interest that steroiodogenic tissues, which express high levels of SR-BI *in vivo*, are endowed with an intricate microvillar system for the trapping of lipoproteins [111,112]. This general region of steroidogenic cells is referred to as the microvillar compartment, and the specialized space created between adjacent microvilli are called microvillar channels; these are specialized domains that form by staking of microvilli or the juxtaposition of microvilli with the plasma membrane. It is the microvillar channels where the various lipoproteins are trapped prior to the selective uptake of CEs into cells [111-113]. Electron microscopic immunocytochemical techniques reveal heavy labeling for SR-BI specifically in these regions (corresponding to such microvilli and microvillar channels) and at present, there is no doubt that issues with microvillar compartments expressing high levels of SR-BI are also active in selective CE uptake [74,75,108-110,114-117]. The formation of these specialized microvillar channels appear to be dependent on the presence of SR-BI since these microvillar are quantitatively reduced in adrenals from SR-BI null mice [118]. Conversely, overexpression of SR-BI promotes microvillar channel formation in both steroidogenic and non-steroidogenic cells *in vitro *[114,116,117]. Additionally, SR-BI has been functionally associated with caveolae/lipid rafts, although this has not always been the case [74,119-124]. SR-BI does show specificity for apolipoproteins, but interacts promiscuously with HDL, LDL, amino acid modified LDL, phospholipids and a variety of other ligands [9,20,105,125,126]. Hormone treatment or other factors which increase the demand for cholesterol also increase the expression of SR-BI and the influx of lipoprotein cholesteryl esters [74,75,108-110,127]. Deletion of the SR-BI gene in mice resulted in increased circulating levels of HDL-cholesterol, substantially reduced stored tissue cholesterol [128,129], and inhibited the selective uptake, storage, and utilization of cholesterol by steroid-hormone producing cells.

#### (ii) SR-BI-mediated selective transport of lipoprotein-derived cholesteryl esters

The mechanism by which SR-BI mediates selective transfer of CE from the cell surface to cell interior is not clearly defined, but may require participation of accessory proteins and lipids, alterations in physicochemical characteristics of the plasma membrane and the physical forms of SR-BI itself. The entire process of "selective" cholesterol ester delivery and its subsequent utilization for steroid synthesis can be broadly divided into three distinct steps, each of which may involve multiple complex processes. The first step in the selective CE uptake is the transfer of lipoprotein-associated CE to the plasma membrane, the second step entails the translocation of CE from the plasma membrane to lipid droplets within the interior of the cell; and the third step in the process is the movement of cholesterol from intracellular lipid droplets to mitochondria for steroid hormone synthesis.

*Events connected with the SR-BI-mediated transfer of lipoprotein cholesteryl esters to the cell surface*--The initial step in the transfer of lipoprotein-cholesteryl esters to the plasma membrane is the binding of cholesterol-rich lipoproteins to the cell surface/microvilli-associated SR-BI followed by release of lipoprotein-cholesteryl esters to the plasma membrane. While SR-BI-lipoprotein interaction is important, it is not sufficient to promote selective transfer of cholesteryl esters to the plasma membrane. This assertion is based on several observations including the fact that mutations of certain glycosylation sites do not affect binding but inhibit selective cholesteryl ester uptake [130]. It has been proposed that SR-BI forms a hydrophobic "channel" through which the cholesteryl esters in SR-BI-associated lipoprotein move down in a concentration gradient manner, and thus, any changes in the structure of the receptor might alter the "channel" without impacting the binding of the lipoprotein [130]. Moreover, it has been suggested that the localization of SR-BI within microvilli or the association of SR-BI with caveolae contributes to the movement of cholesteryl esters into these specialized regions of the plasma membrane.Furthermore, these specialized microvilli/microvillar channel regions and/or caveolae could increase functional efficiency of the transfer process through increased availability of donor particles [111-113]. Limited studies have also suggested the involvement of C-terminal and extracellular domain (ECD) domains of SR-BI in the selective cholesterol uptake process [131-134]. Finally, evidence is accumulating suggesting that accessory proteins (see below) and lipids, in addition to SR-BI, contribute to the selective uptake process [135-145].

*SR-BI interacting accessory proteins*--other significant progress in the area of SR-BI structure and function is the realization that accessory proteins may interact functionally with SR-BI and facilitate the dimerization process, enhance selective HDL-CE uptake and promote cell surface architectural changes. In recent years, one such protein, the PDZ domain containing protein called CLAMP, has been identified with SR-BI mediated selective CE uptake [127]. (The name PDZ is derived from the first three proteins in which these domains were found: PSD-95 Dlg, and ZO1; these domains range 70-90 amino acids in length and recognize 3-5 residue motifs that occur at the C-terminus of target proteins or structurally related internal peptide motifs [146-152]). CLAMP was purified from rat liver extracts by affinity chromatography using the last 15 amino acids of the carboxyl terminus of SR-BI, and co-expression of CLAMP, (which is identical to and now referred to as PDZK1 or NHERF3 [127,141,153,154]) and SR-BI in CHO cells led to a two-fold increase in selective CE uptake. More recently, Silver by using transgenic animals expressing SR-BI with a mutated PDZK1 interacting domain provided evidence that PDZK1 interacting domain of SR-BI is essential for cell surface expression of hepatic SR-BI *in vivo *[136]. Also, an endogenous regulator of PDZK1, termed small PDZK1-associated protein (SAP, DD96/MAP17) has been characterized, which when overexpressed in the liver causes increased degradation of PDZK1, resulting in hepatic SR-BI deficiency and markedly increased plasma HDL cholesterol [137].

Krieger and colleagues further demonstrated that targeted disruption of the PDZK1 gene induced hypercholesterolemia, and resulted in substantial reduction of hepatic and intestinal SR-BI, without affecting SR-BI or cholesteryl ester stores in steroidogenic organs [138]. The latter observations are in agreement with the results showing that adrenal and gonads express very low levels of PDZK1 as compared to the liver and strongly suggest the possibility that different types of PDZ-domain containing proteins impact SR-BI in a tissue-specific manner. Interestingly, hepatic expression of SR-BII, a variant with an alternate C-terminal domain, is not affected in PDZK1 knockout mice, suggesting that PDZK1 specifically regulates SR-BI expression and function in the liver. Further studies demonstrated that overexpressing full-length PDZK1 in PDZK1 null mice restored normal hepatic SR-BI protein levels [155]. Likewise, hepatic overexpression of wild-type SR-BI restored near or greater than normal levels of functional, cell surface SR-BI protein levels in the livers of SR-BI(-/-)/PDZK1(-/-) double knock-out mice and such genetic manipulation restored normal lipoprotein metabolism in the absence of PDZK1 [156]. From these studies, it is concluded that PDZK1 is important for maintaining adequate steady state levels of SR-BI in the liver but is not essential for cell surface expression or function of hepatic SR-BI [155,156]. More recent studies indicate that hormone (glucagon)-mediated phosphorylation of the C-terminal region of PDZK1 as well as the presence of all four PDZ domains in PDZK1 is required for normal abundance, localization and, therefore, function of hepatic SR-BI [157,158]. More recently, Komori *et al*, using a transgenic mouse model coexpressing both CLA-1 (human homolog of SR-BI), and human PDZK1 provided evidence that PDZK1 is also an important enhancer of CLA-1 expression in the liver [159]. Besides the liver, a role for PDZK1 is indicated in HDL/SR-BI signaling in endothelium and in the maintenance of endothelial monolayer integrity [142].

Given that PDZK1 is not expressed in steroid producing tissues, coupled with the demonstration that PDZK1 null mice show normal expression of SR-BI in the adrenal gland and ovary, we considered the possibility that additional PDZ domains with specificity for other PDZ proteins may be involved in the regulation of SR-BI function in steroidogenic tissues. We searched for and identified additional Class **I, II **and **III **PDZ interacting domains in the *C*-terminal cytoplasmic tail of SR-BI (Table 2) using a simple modular architecture research tool http://SMART.embl-heidelberg.de. To a large extent these PDZ domains are well conserved among various mammalian species (i.e., mouse, rat, hamster, pig, bovine and human SR-BI), and the Class II PDZ-domain (PDZK1 site) described above is simply the final extreme end site on the SR-BI, which is not well conserved. The function of the other sites is as yet unknown, but it is of interest that the C-terminal domain of SR-B**II **(the alternative spliced form of SR-BI) lacks terminal PDZ domain and contains entirely different sets of PDZ interacting domains while another family member, CD36 contains no PDZ sites. Interestingly both SR-BII and CD36 are less efficient in mediating selective HDL-CE uptake as compared to SR-BI [131,132]. We believe these SR-BI PDZ-domain binding motifs may, in fact, be important for steroidogenic tissues--in that they permit binding to a variety of PDZ-containing proteins [146-151]. Indeed, our preliminary Protein Array analysis indicated significant interaction between hCLA-1/SR-BI and PDZ-domain(s) of RGS12, CLP36 (also called hCLIM1 or elfin), RIL, PSD-95, and Mint-3-proteins [160-172]. Among these, RGS12 and RIL, PSD-95 PDZ proteins are known to be highly expressed in steroidogenic proteins [160,163,170]. In addition, PDZ-RhoGEF, a novel guanine nucleotide exchange factor (GEF) for Rho-like proteins, contains a PDZ domain which shows high affinity for the actin cytoskeleton, and is also highly expressed in various steroidogenic tissues [173,174].

**Table 2:** Potential Consensus PDZ Domain Binding Sequences in SR-BI, SR-BII and CD36

Mouse SR-BI:	QLRSQEKCF**LFW**SGSKKGSQDK**EAI**QAYS**ESL**MS**PAA**KG**TVL**QE**AKL**
	QLRSQEKC**FLF**WSGSKKGSQDKEA**IQA**YSESL**MSP**AAKGTVLQEAKL
Rat SR-BI:	QLRSQEKCF**LFW**SGSKKGSQDK**EAM**QAYSWSLMS**PAA**KG**TVL**QE**AKL**
	QLRSQEKC**FLF**WSGSKKGSQDKEA**MQA**YSWSL**MSP**AAKGTVLQEAKL
Hamster SR-BI:	QLRSQEKCF**LFW**SGSKKGSQDK**EAI**QAYA**ESL**MS**PAA**KG**TVL**QE**AKL**
	QLRSQEKC**FLF**WSGSKKGSQDKEA**IQA**YAESL**MSP**AAKGTVLQEAKL
Rabbit SR-BI:	QVRSQEKCY**LFW**SGSKKGSKDK**EAI**QAYS**ESL**MT**PDP**KG**TVL**QE**ARL**
	QVRSQEKCYLFWSGSKKGSKDKEA**IQA**YSESL**MTP**DPKGTVLQEARL
Pig SR-BI:	QIRSQEKCY**LFW**SSSKKGSKDK**EAI**QAYS**ESL**MT**PAP**KG**TVL**QE**ARL**
	QIRSQEKCYLFWSSSKKGSKDKEA**IQA**YSESL**MTP**APKGTVLQEARL
Cow SR-BI:	QIRSQEKCY**LFW**ISFKKGSKDK**EAV**QAYS**EFL**MTSPPKG**TVL**QE**ARL**
	QIRSQEKCYLFW**ISF**KKGSKDKEA**VQA**YSEFLMTSPPKGTVLQEARL
Human SR-BI:	QIRSQEKCY**LFW**SSSKKGSKDK**EAI**QAYS**ESL**MTSAPKG**SVL**QE**AKL**
	QIRSQEKCYLFWSSSKKGSKDKEA**IQA**YSESLMTSAPKGSVLQEAKL
Mouse SR-BII:	QLRSQGPE**DTI**SPPNLIAWSDQPPSPYTPLLEDSLSGQPTS**AMA**
	QLRSQGPEDTISPPNLIAWSDQPPSPYTPLLEDSLSGQPTSAMA
Rat CD36:	RSKNGK-None

PDZ Domain-Class I: S or T-X-I, V, L or M; PDZ Domain-Class II: **ψ-X-ψ; PDZ Domain-Class III: D or E -X-ψ**. The C-terminal residue is referred to as Po residue; subsequent residues towards the N-terminus are termed P-1, P-2, P-3 etc. **X **denotes any amino acid (no specificity defined at this position for this class). **ψ **denotes a hydrophobic amino acid usually V, I or L. The sequences of C-terminal domain of SR-BI, SR-BII and CD36 are presented in duplicates to accomodate 3 letter overlapping combinations of putative PDZ binding sites. The putative PDZ domain binding sequences (three letters) are shown as bold letters.

Additionally, using a transient overexpression strategy, we directly examined the effect of a number of PDZ domain containing proteins on SR-BI-mediated selective HDL-CE uptake in representative steroidogenic (MLTC, mouse testicular Leydig cells) and hepatic (HepG2, human hepatoma cells) cell lines. Co-transfection with PDZ proteins, DLG3, DLG5, or PDLIM1 plus SR-BI significantly increased selective HDL-CE uptake in both HepG2 and MLTC cells as compared to cells transfected with SR-BI alone. Moreover, several additional PDZ proteins such as GOPC, HTRA2, INADAL, LIN7B, MAG12, MAG13, MAST2 and PARD6B variably, but significantly enhanced selective HDL-CE uptake. In contrast, expression of other PDZ domains containing proteins including DVL1, DLV3, LIN7C, MPP2, DLG2, DLG3, or GRIP1 showed no demonstratable effect on SR-BI-mediated selective HDL-CE uptake. These data led us to conclude that steroidogenic cells, like hepatocytes, require the participation of PDZ type proteins for the maximal functional efficiency of SR-BI, but show a wide specificity towards PDZ proteins. However, at present, a number of important questions remain unanswered. For example, it is unclear how these various proteins regulate SR-BI function, whether they are expressed in steroidogenic cells, and their expression, like SR-BI, is regulated by tropic hormones and whether different PDZ proteins differentially impact SR-BI function in different steroidogenic cell types (i.e., adrenal, ovarian and testicular cells).

*SR-BI-mediated alterations in the lipid composition of plasma membranes*--It has been suggested that SR-BI may alter the composition of lipid domains of plasma membranes which then leads to changes in free cholesterol flux, changes in membrane cholesterol content, changes in plasma membrane phosphatidylcholine subspecies or in altered physical/chemical properties of the membrane [143,144]. In another study, Chen *et al*. reported that expression of SR-BI in RAW macrophages markedly reduced ABCA1-mediated cholesterol efflux to apolipoprotein A1 presumably by sequestering cholesterol that is normally available to ABCA1 for efflux [145]. On the other hand, it is demonstrated that sphingomyelin and ceramide in the lipoproteins and the cell membranes regulate the SR-BI-mediated selective uptake of CE in SR-BI transfected CHO cells, hepatocytes (HepG2) and adrenocortical cells (Y1BS1), possibly by interacting with the sterol ring or with SR-BI itself [140]. Our recent studies suggest that SR-BI may also be involved in the regulation of cell surface expression of microvillar channel formation, a function that may increase the functional efficiency of the selective CE uptake process through increased trapping and binding of HDL at the cell surface [114,116,117].

*SR-BI dimerization*--the physical form of SR-BI may also play an important role in its ability to mediate selective CE transport. Indeed, it is becoming increasingly clear that hormone-induced changes in tissues--which alter the expression of SR-BI, alter selective CE uptake in the same tissues, and correspondingly produce architectural changes in the cell surface of affected cells--also show changes in 'dimerization' of SR-BI in cell or tissue samples (for simplicity, we use the term dimerization here to include the multiple forms of the SR-BI protein; i.e., dimers, and higher order oligomers). In one of the earliest direct demonstrations of protein-protein interaction involving SR-BI, our laboratory demonstrated (by SDS PAGE-Western blotting) that SR-BI exists as homodimers in 17α-ethinyl estradiol (17α-E_2_) primed and microvilli-enriched rat adrenal plasma membrane [109]. In subsequent studies, we were able to demonstrate that SR-BI exists in dimeric and high order oligomeric forms in all cells and tissue which are active in 'selective' uptake of HDL-CEs (e.g., hormone activated steroidogenic tissues such as mouse adrenal, testis, and ovary; steroidogenic cells such as rat ovarian luteal cells, Y1-BS1 mouse adrenocortical cells, R2C rat Leydig cells, and MLTC mouse Leydig cells; liver from SR-BI transgenic mice; SR-BI overexpressing non-steroidogenic cells such as HEK 293, Y1-BS1, CHO and COS cells; Sf9 insect cells programmed to express rat SR-BI [114,116,117]. Early functional evidence for SR-BI dimerization came from the observation that in normal rat adrenal tissue, SR-BI exists primarily in the monomeric form with some dimer formation. ACTH stimulation increased the dimerization of SR-BI in this tissue along with increased selective CE uptake, and dexamethasone-induced loss of ACTH led dramatically to the loss of SR-BI, SR-BI dimers and selective HDL-CE uptake [109,116]. These results are coupled with striking architectural changes of the microvillar compartment at the adrenocortical cell surface, and suggest that SR-BI dimers may, in a very basic way, be associated with SR-BI sites of action and function.

**Figure 4:**
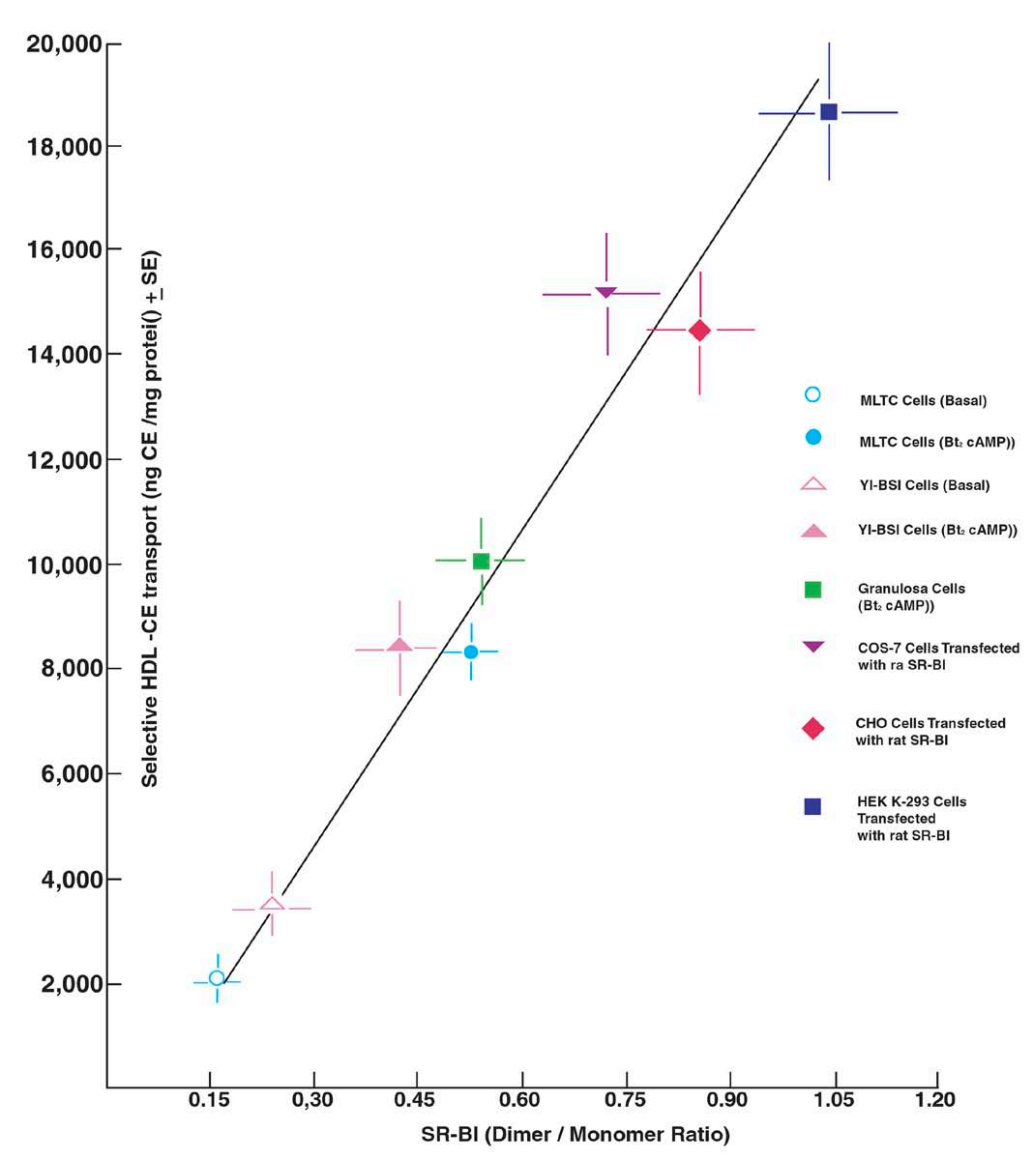
**Correlation between the cellular levels of SR-BI dimers and the functional efficiency of selective HDL-CE uptake**. Appropriate Western blots from various cell types were scanned for SR-BI monomers and dimers and dimer/monomer ratios were plotted against the respective selective HDL-CE uptake data. The results show that dimer/monomer ratios determined for individual cell types correlate significantly with their respective SR-BI-mediated selective HDL-CE uptake.

Additional functional evidence came from our laboratory showing a strong correlation from the levels of SR-BI dimers and increased selective HDL-CE uptake in cells and tissues (Fig. 4), and from co-immunoprecipitation studies of epitope-tagged SR-BIs (SR-BI-cMyc and SR-BI-V5) used to demonstrate that SR-BI can exist as homodimers [116]. The use of cross-linking agents further confirmed that SR-BI forms dimers in native steroidogenic cell lines (endogenous), as well as in a heterologous insect cell expression system [114]. Also, analysis of cellular extracts from SR-BI transfected HEK-293 cells or ACTH-treated Y1-BS1 cells by size-exclusion chromatography and sucrose density centrifugation demonstrated that a significant portion of SR-BI exists in dimeric and oligomeric forms. As an independent measure, we have utilized immunoelectron microscopy which further provides convincing evidence for the formation of SR-BI:SR-BI homodimers. We showed that when double tagged-SR-BI proteins (SR-BI-cMyc and SR-BI-V5) are co-expressed in HEK-293 cells and the different proteins are subsequently immunostained and identified with two differently stained gold particles, there is mixing and clustering of gold particles suggesting 1) that the proteins travel to the same cell location, and 2) that many of the gold particles are in exceedingly close physical contact, i.e., within the distance accepted for protein dimers by fluorescent resonance energy transfer (FRET) technique [116,117]. Similar results were obtained when Y1-BS1 mouse adrenocortical cells were transfected with V5 and/or cMyc tagged-SR-BI proteins. Interestingly, SR-BI transfected Y1-BS1 demonstrated major architectural changes along with the formation of double membranes in flower like arrangements. Gold-labeled secondary antibodies against V5 or cMyc antibody localized SR-BI to these sites, and revealed substantial dimer formation of this protein--shown by close contact between gold particles [116,117].

From the above discussion it is apparent that while the understanding of the functional significance of SR-BI dimerization in steroidogenic tissues and cell lines which utilize the selective pathway for cholesterol transport is improving, the structural basis of the intramolecular interactions involved in SR-BI dimerization and function is not completely understood. In particular, the information about the contribution of the extracellular domain (ECD) of SR-BI either independently or in cooperation with the *C*-terminal domain on SR-BI dimerization, SR-BI-induced microvillar channel formation, and selective HDL-CE uptake remains sketchy. In an effort to further expand our understanding about the structure-function relationships and dynamics of SR-BI activity, we recently carried out studies aimed at determining the structural and functional contributions of cysteine residues within the SR-BI. We focused our efforts on cysteine residues because: (a) cysteine residues are integral for inducing and maintaining the three-dimensional confirmation in proteins by forming critical inter- and intra-molecular disulfide bond linkages; (b) sulfhydryl (SH) side chains of cysteins are polar similar to that of the hydroxyl group (OH) of serines and can participate in hydrogen bonding interactions and facilitate protein-protein interactions; (c) cysteine side-chains are preferred sites for various biological coupling and conjugation reactions such as palmitoylation, isoprenylation, disulfide cross-linking, and thiol-disulfide exchange which are known to play critical roles in intracellular protein trafficking, stability and/or activity; and (d) the SR-BI contains several cysteine residues that are highly conserved across the species and uniquely distributed within the different domains of the SR-BI molecule and as such are highly likely to contribute towards SR-BI structure and function [175-179].

We chose to study the rat SR-BI because it contains more conserved cysteine sequences than SR-BI from any other species. Its sequence contains a total of eight cysteine (C) residues (C21, C251, C280, C321, C323, C334, C384, and C470). With the exception of C21, the remaining seven residues are highly conserved in other species including the mouse, hamster, rabbit, pig, cow, dog, tree shrew and human. Five residues (C280, C321, C323, C334, and C384) are clustered in the C-terminal half of the putative extracellular domain (ECD). The remaining three cysteine residues are equally distributed in the N-terminal transmembrane domain (C21), N-terminal half of the ECD (C251), and the C-terminal domain (C470). Given that the extracellular domain contains six conserved cysteine residues, these could form up to three disulfide bonds, which in turn could help to stabilize the confirmation of SR-BI or participate in its dimerization. We replaced these cysteine residues with serine (S) singly or in pairs, expressed the mutated SR-BI constructs in CHO or COS-7 cells and examined the impact of these mutations on SR-BI expression and function. Overall, these studies indicated that C280S, C321S, C323S and C334S residues of the extracellular domain (ECD) are necessary for preserving normal SR-B (HDL) binding activity, selective CE uptake, and/or cell surface expression. Interestingly, mutation of any of these four cysteine residues to serine resulted in a robust induction of SR-BI dimer formation, but they are rendered non-functional because these residues are most likely also essential for the optimal HDL binding and hence, the selective CE uptake.

#### (iii) Translocation of cholesteryl esters/cholesterol from cell surface (plasma membrane) to lipid droplets

Although selective uptake of cholesteryl esters for all practical purposes is considered to be non-endocytic, at least from the point of view of uptake of the intact lipoprotein particle, there are controversies regarding cholestryl ester movement to lipid droplets. However, there are some suggestions that HDL-cholesteryl esters are delivered to the cell interior by the retero-endocytosis, where the receptor-bound HDL particle analogous to the transferrin receptor system is internalized, traverses an intracellular pathway during which cholesteryl esters are transferred to the cell interior and the HDL particle is recycled back to the plasma membrane where the lipid depleted HDL is now released [180-185]. This possibility appears to be weak given the overwhelming morphological evidence both at the light- and electron microscopic evidence showing that *in vivo *and in vitro HDL-cholesteryl ester delivery to adrenal and ovarian luteal tissues and cultured cells, respectively, does not involve internalization of the intact HDL particle itself, [73,90,100,101,103,186,187]. It is possible that a small amount of HDL internalization in cultured cells reported by some investigators was in fact due to non-specific endocytosis of the HDL particle; indeed, there is considerable in vitro evidence that cultured cells can internalize a variety of receptor ligands in a non-specific manner [188-191]. It has also been suggested that HDL- cholesteryl esters are delivered to intracellular membranes via the formation of complexes with caveolin, annexin and cyclophilins [192]. In this regard, it is noteworthy that caveolin is a component of several intracellular vesicle populations, caveolin-1 is required for lipid droplets formation, and all forms of caveolins (i.e. caveolin-1, -2 and -3) can associate with lipid droplets [193-197].

Lipid droplets are associated with proteins involved in vesicle-vesicle targeting and the fusion process in the cells including *N*-ethylmaleimide (NEM)-sensitive factor (NSF), soluble NSF attachment protein (alpha-SNAP), and the SNAP receptors (SNAREs), synaptosomal-associated protein of 23 kDa (SNAP23), syntaxin-5 and vesicle-associated membrane protein 4 (VAMP4), a chaperone protein that participates in [198]. In this context our own studies have shown that treatment of steroidogenic cells with NEM, an inhibitor of NSF, results in a total block of HDL-derived selective cholesteryl ester uptake [72,73]. Thus, intracellular transport of cholesteryl esters to lipid droplets might involve active participation of carrier- and/or vesicle-mediated cholesterol transport processes. On the other hand, ~75% of SR-BI delivered HDL-cholesteryl esters were reported to be hydrolyzed by non-lysosomal neutral cholesteryl ester hydrolases (nCEHs) suggesting that freshly delivered cholesteryl esters could also be transported in the form of free cholesterol to various cellular destinations [199]. Existing literature also supports this possibility given that fatty acid composition of cholesteryl esters in the rodent adrenal and ovary is significantly different from that of plasma or HDL, i.e., this could only occur if internalized plasma lipoprotein-derived cholesterol esters were at first hydrolyzed and then ensuing free cholesterol re-esterified with fatty acids to a defined fatty acid composition that is unique and specific for each of the steroidogenic tissues [200-203]. A combination of vesicular and non-vesicular transport processes most likely facilitates the transport of the newly released free cholesterol to the ER for its esterification and subsequent storage in lipid droplets [55,57,66,204]. Depending on cellular needs, free cholesterol could also be redirected to the plasma membrane or mitochondria (for steroid synthesis), again possibly via vesicular and/or non-vesicular transport pathways [55,57,66,204].

### Intracellular cholesterol processing and its transport to mitochondria for the initiation of steroid synthesis

Steroid producing cells through the use of multiple cholesterol supply sources discussed above maintain adequate cholesterol reserves primarily in the form of lipid droplets that enable them to quickly respond to tropic hormone stimulation with the rapid mobilization of cellular cholesterol reserves and ensuing transport to mitochondria for steroidogenesis. In adrenal and ovarian cells, cellular stores of cholesterol esters are constantly replenished by the delivery of plasma cholesterol through endocytic or selective pathway (depending on species and lipoprotein type), whereas this chore in Leydig cells, under normal physiological conditions, is mainly achieved through increased *de novo *cholesterol synthesis. During acute hormonal stimulation, these endogenously stored cholesterol esters are rapidly mobilized (hydrolyzed) and released free-cholesterol is efficiently transported to and within the mitochondria for its conversion to pregnenolone, the precursor of all steroid hormones. This entire process involving the intracellular cholesterol mobilization, processing and transport to the appropriate site within the mitochondria for side-chain cleavage and pregnenolone production can be broadly divided into two separate, but equally important segments: a) mobilization of cholesterol from intracellular stores, particularly from lipid droplets; b) transport of mobilized cholesterol to the outer mitochondrial membrane; and c), transfer of this cholesterol from the outer to the inner mitochondrial membrane. In the following sections, we will discuss characteristics of these three segments of intracellular cholesterol transport and also summarize current understanding about the functional roles of key proteins and factors involved in the mobilization of cellular cholesteryl esters, intracellular transport of newly released cholesterol to the outer mitochondrial membrane and its subsequent translocation to the inner mitochondrial membrane for the initiation of steroidogenesis.

### (a) Mobilization of cellular cholesterol reserves in response to acute hormonal stimulation

It is well known that adrenal, ovarian and testicular Leydig cells' cholesteryl esters are rapidly depleted following tropic hormone (ACTH, LH/hCG) treatment supporting the notion that mobilization of lipid droplets (LD) stored cholesteryl esters provides cholesterol for acute hormonal stimulation of steroid synthesis [205-207]. This mobilization of substrate cholesterol occurs through tropic hormone-mediated increased formation of second messenger, cAMP followed by activation of PKA, and PKA-mediated phosphorylation (activation) of neutral cholesteryl ester hydrolase (nCEH), resulting in rapid hydrolysis of cholesteryl esters [21-24,24,78-80,205-210]. We reported that hormone-sensitive lipase (HSL) is responsible for the vast majority, if not all, of nCEH activity in the adrenal [208]. This was based on the observation that inactivation of HSL resulted in the loss of >98% of nCEH [208]. Moreover, we reported that adrenocortical cells isolated from HSL null mice show almost complete inhibition of ACTH stimulated and HDL-supported corticosterone secretion (>99%) as compared to cells isolated from control mice, further demonstrating the importance of HSL in adrenal steroidogenesis; HSL null mice also show an increased lipid accumulation in the adrenals and a blunted corticosterone secretion *in vivo *[209,210]. Current evidence also suggests that HSL is likely to function as a cholesteryl ester hydrolase in the ovary [211-213]. There is also a testis-specific isoform of HSL with a molecular mass of 120 kDa [80-82]. This isoform differs from a more common isoform of HSL, which is expressed in adipose tissue and adrenal, ovary and other tissues/cells, by containing some additional 300 amino acids [213-215]. This 120 kDa isoform is predominantly expressed in germ cells of the testis and its expression is hormonally regulated [216]. However, the identity and expression of HSL in testosterone producing testicular Leydig cells has yet to be established.

### (b) Transport of mobilized cholesterol to the outer mitochondrial membrane

The newly released cholesterol is transported to the outer mitochondrial membrane (OMM) for the production of steroid hormones. Because cholesterol is a hydrophobic molecule and diffuses poorly in an aqueous environment, it can traverse from the cytoplasmic locations to the OMM by several potential mechanisms [54-57,66]. Cholesterol can be transported via the vesicular transport mechanism, i.e., it can be incorporated into the vesicular structures involved in cellular trafficking (e.g., transport vesicles, endosomes, and secondary lysosomes) which then fuse either directly or indirectly (through other intermediary membranes) fuses with mitochondria and deliver their cargo to the OMM. However, this pathway appears to play a minor role [55,84]. Cholesterol may also be delivered to OMM via protein-protein interactions between the lipid droplets and mitochondria. As early as in 1975, electron microscopic observations provided evidence suggesting that lipid droplets become juxtaposed during stimulation by tropic hormone [217]. In the last few years, additional evidence has emerged showing potential interactions between lipid droplets and cellular organelles including mitochondria in several cell systems [69,218-220]. More recently, Boström *et al *reported the presence of some constituent proteins of the SNARE complexes on the lipid droplets [198]. (SNARE complexes facilitate fusion between transport vesicles and target membranes during protein trafficking) [221-223]. These proteins include, NSF, α-SNAP, and SNAREs, SNAP23, syntaxin-5, and VAMP4. The authors of this report also provide evidence that VAMP4, syntaxin5 and SNAP23 are required for lipid droplet fusion [198]. More recently, another report provided direct evidence showing that the SNAP23 protein promotes interaction between lipid droplets and mitochondria [224]. Other reports suggest that steroidogenic cells express high-levels of some members of SNARE proteins such as Syntaxin-17 SNAP23, and SNAP25, and that expression of the neuronal type of SNAP25 is hormonally regulated in ovarian granulosa cells [225-229]. These various observations strongly suggest that SNARE proteins may mediate the transport of cholesterol substrate from lipid droplets to steroidogenic mitochondria, most likely by promoting the functional interaction between lipid droplets and mitochondria.

A second potential mechanism by which mobilized cholesterol from lipid droplets may be delivered to the mitochondrial for steroid synthesis is through a non-vesicular transport process involving high-affinity cholesterol binding proteins [55,57,66,84,230]. Earlier studies indicated that sterol carrier protein_2 _(SCP_2_), a nonspecific lipid transfer protein, mediates cholesterol transport to steroidogenic mitochondria and also stimulates steroid hormone biosynthesis [230-232]. Contrary to these findings, more recent metabolic and genetic evidence suggests that SCP_2 _mainly functions as a carrier for fatty acyl CoAs, facilitates branched-chain fatty acid oxidation and regulates the distribution of key lipid signaling molecules (e.g., FA, fatty acyl CoAs, LPA, PI and sphingolipids) between lipid rafts/caveolae and intracellular sites, while it appears to play a minor role in cellular cholesterol trafficking primarily because of its very low affinity for cholesterol [233,234]. More recently, Breslow's laboratory has identified a subfamily of lipid binding proteins referred to as StarD4, StarD5 and StarD6 (StarD4 subfamily) [235,236], which are structurally related to steroidogenic acute regulatory protein (StarD1/StAR), a prototype of the steroidogenic acute regulatory-related lipid transfer (START) domain containing a superfamily of proteins [84,235-238]. StarD4 and StarD5 are widely expressed in steroid producing cells, while StarD6 expression appears to be mostly restricted to the testicular germ cells [239,240]. In contrast to StarD1 and StarD3/MLN64, StarD4, StarD5 and StarD6 lack any signal peptides, and thus, they are not targeted to any specific cellular organelles. Therefore, they are considered to be a cytosolic protein like StarD2/PCTP [238,239]. Interestingly, StarD6 despite lacking any *N*-terminal target sequences that should direct this protein to mitochondria is reported to have physicochemical properties and biological activity (stimulation of steroidogenesis) similar to that of StarD1/StAR, while StarD4 and StarD5 exhibit low levels of StarD1/StAR-like activity [240]. Both StarD4 and StarD5, however, bind free cholesterol with high-affinity and specificity, facilitate cholesterol transport through an aqueous environment and have been shown to play important roles in the maintenance of cellular cholesterol homeostasis [241,242]. The ability and specificity of StarD4 and StarD5 to bind cholesterol, coupled with their high levels of expression in steroidogenic tissues, raises the strong possibility that StarD4 and StarD5 facilitate cholesterol transport to the outer mitochondrial membrane. However, confirmation of this possibility must await the relevant experimental evidence.

Extensive but mostly circumstantial evidence suggests that cellular architecture and cytoskeletal elements, in particular, vimentin-intermediate filaments (IF, Type III) may also be involved in facilitating cholesterol transport to mitochondria [8,243,244]. Vimentin-intermediate filament constitutes part of the network of the cytoskeleton [245]. It is expressed in many cell types including adrenal, ovarian and testicular Leydig cells [245-250]. Several different reports of proteomic analyses of lipid droplets isolated from cells have consistently identified vimentin as a lipid droplet associated protein [251-253]. Vimentin has been shown to interact with several different proteins, including some motor-like propertiesand sterol binding properties [254-256]. Using a proteomics approach, vimentin was identified as an interacting partner of agonist stimulated β_3_-adrenergic receptors and this interaction was shown to be important for activation of ERK and stimulation of lipolysis, providing the additional involvement of vimentin in lipid droplet metabolism [257]. The overexpression of ORP4, which interacts with vimentin and causes its aggregation, results in a defect in cholesterol esterification [254]. Likewise, adrenal cells lacking vimentin display a defect in the re-esterification of LDL cholesterol without any alterations in LDL-receptor-mediated endocytosis [258]. Hall and colleagues reported a close association of both functional mitochondria and cholesterol-enriched lipid droplets with the intermediate filaments in Y1 adrenal tumor cells, and testicular Leydig cells and suggested the possibility that such binding may facilitate the transport of cholesterol to mitochondria for steroid synthesis [248-250]. Furthermore, binding of lipid droplets and mitochondria to vimentin-intermediate filaments may also provide an ideal platform for docking of lipid droplets to the mitochondria and secondarily increased cholesterol transport to mitochondria. Besides morphological evidence, a number of biochemical studies employing pharmacological inhibitors raised the possibility that cytoskeletal elements including vimentin may contribute to the cholesterol transport to mitochondria and the regulation of steroidogenesis although conflicting results have been generated [243]. Also, it is important to realize that agents which were previously used in many of these studies to disrupt microfilaments, e.g., nocadazole, cytochalasin, and cyclohexamide, affect tubulin and actin, but have no effects on vimentin [259]. Obviously, more experimental work is needed to clearly define the role of cytoskeletal elements/structures including vimentin intermediate filaments in cholesterol transport to mitochondria and regulation of steroidogenesis.

### (c) Translocation of cholesterol from the outer mitochondrial membrane to the inner mitochondrial P450scc site

The second critical step in steroid hormone biosynthesis is delivery of the cholesterol substrate to the inner mitochondrial membrane (IMM) sites, where cholesterol side-chain cleavage P450scc is located, and the enzyme that catalyzes the conversion of cholesterol to pregnenolone takes place [3-6,8]. This step is rate-limiting because the hydrophobic cholesterol cannot freely diffuse through the aqueous intermembrane space of the mitochondria to support acute steroid synthesis and requires the participation of a *de novo *synthesized labile protein [8,47-50,260-264]. This putative labile protein evaded detection for almost twenty years until 1983 when Orme-Johnson's laboratory first demonstrated that acute ACTH stimulation of adrenocortical cell steroidogenesis was accompanied by a rapid induction of 37 kDa phosphoprotein [265]. Subsequent studies from her laboratory provided further characterization of this phosphoprotein in the adrenal and also demonstrated its presence and hormonal induction in corpus luteum and testicular Leydig cells [266-272]. Stocco and colleagues confirmed these observations in MA-10 Leydig tumor cells, and subsequently cloned this protein and named it steroidogenic acute regulatory protein (StAR) [273,274]. StAR has been cloned from many species and is highly conserved across the species [275]. StAR protein possesses all of the necessary characteristics of the acute regulator of steroid synthesis in steroidogenic cells i.e., its synthesis is specifically induced in steroidogenic cells of the adrenal and gonads in response to tropic hormone, is highly labile, and is sensitive to the protein synthesis inhibitor, cycloheximide [8,23,84,264-273].

The role of StAR protein in the regulation of acute hormonal steroidogenesis was supported by three lines of evidence. First, transfection of a model testicular Leydig cell line (MA-10 cells) with a StAR plasmid stimulated steroid production to the same extent as that seen with a maximum stimulating dose of cAMP analog [274]. Second, co-transfection of StAR plus a fusion protein complex of P450scc plasmids in a heterologous cell system produced several-fold more pregnenolone (steroid) as compared to cells transfected with P450scc fusion complex alone [276,277]. Third, the most compelling evidence for a role of StAR in steroiodogenesis was provided by demonstrating that mutations in the StAR gene cause a fatal condition in newborns, the congenital lipoid adrenal hyperplasia (lipoid CAH), characterized by severe impairment of steroiodogenesis, hypertrophied adrenals containing high levels of cholesterol esters and free cholesterol and increased amounts of neutral lipids in the testicular Leydig cells [276,278]. Depletion of the murine StAR gene by homologous recombination yielded an identical phenotype of impaired steroidogenesis and lipid accumulation in the adrenal and gonads [279,280].

In accordance with its role in the acute regulation of steroidogenesis, StAR is expressed mainly in the adrenal cortex, steroid producing cells of the ovary and testicular Leydig cells [277,281]. Significant expression of StAR is also reported in the rodent brain cell type that parallels the expression of P450scc and other steroidogenic enzymes, but its potential role in neurosteroidogenesis is not yet established [84]. In contrast, StAR expression is not detected in another major steroidogenic tissue, the placenta, which secretes progesterone constitutively [277,281]. StAR is synthesized as a short-lived cytoplasmic 37-kDa protein with a mitochondrial targeting peptide that is cleaved upon mitochondrial import to yield the long-lived intramitochondrial 30-kDa form [84,269,273]. StAR functions as a sterol transfer protein, binds cholesterol, mediates the acute steroidogenic response by moving cholesterol OMM to IMM, acts on the OMM, and requires structural change previously described as a pH-dependent molten globule [282-287]. StAR is also a prototype of a family of proteins that contain StAR-related lipid transfer (START) domains (StarD proteins), of which StarD3/MLN64, StarD4, 5 and 6 exhibit steroidogenic potential [235,236,238,240,282-287].

Given that StAR (StarD1) acts on the outer membrane in mediating the transfer of cholesterol from the OMM to the IMM, and raises the possibility that it may be a component of a multi-protein complex [84,284-289]. Several lines of evidence indicate that peripheral-type benzodiazepine receptor (PBR) is also involved in mitochondrial import of cholesterol substrate [290,291]. PBR, which is now referred to as translocator protein (18 kDa, TSPO) is a high-affinity drug- and cholesterol-binding mitochondrial protein, with a cytoplasmic domain containing a cholesterol recognition amino acid consensus (CRAC) domain [292]. TSPO is expressed ubiquitously in the OMM, but is more abundant in the adrenal gland and steroidogenic cells of gonads [288,289,291-296]. TSPO ligands stimulate steroid synthesis and promote translocation of cholesterol from OMM to the IMM in testicular Leydig cells, ovarian granulosa cells, and adrenocortical cells [290,291,293-298]. Mutagenesis of the CRAC domain interferes with cholesterol binding and transfer of cholesterol to IMM [299,300]. Targeted deletion of the TSPO gene in a Leydig cell line (TSPO-deficient R2C cells) blocked cholesterol transport into the mitochondria and dramatically reduced steroid production, whereas reintroduction of TSPO in the deficient cell line restored the steroidogenic capacity [301]. TSPO is a component of a 140-200 kDa multi-protein complex consisting of 18-kDa TSPO itself (and its polymorphic form), the 34-kDa voltage-dependent anion channel (VDAC), the 30-kDa adenine nucleotide translocator (ANC), a 10-kDa protein (pk 10), TSPO-associated protein-1 (PRAX-1), and the TSPO and protein kinase A (PKA) regulatory subunit RIα-associated protein (PAP7) [288].

Increasing evidence now suggests that TSPO and StAR interact functionally in mediating the transfer of cholesterol from the outer mitochondrial membrane to the inner mitochondrial membrane. For example, FRET measurements indicated that StAR and TSPO come within the 100 A° of each other consistent with the possibility that StAR and TSPO may interact with each other [302]. However, using a complementary bioluminescence resonance energy transfer, the same laboratory was unable to provide evidence for protein-protein interaction between TSPO and StAR [303]. Hauet *et al *provided evidence that isolated mitochondria from Tom20/StAR overexpressing MA-10 Leydig cells produced steroids at a maximal level, but these cells lose their steroidogenic capacity if exposed to TSPO-antisense oligonucleotide [304]. Interestingly, re-introduction of recombinant TSPO into the mitochondrial environment in vitro restored the steroidogenesis [304]. The current thinking is that functional cooperation between proteins such as the cAMP-dependent protein kinase regulatory subunitα (PKA-RIα) and the PKA-RIα- and TSPO-associated acyl-coenzyme a binding domain containing 3 (ACBD3) proteins, PAP7, cholesterol is transferred to and docked at the OMM [288]. The TSPO-dependent import of StAR into mitochondria, StAR interaction with VDAC1, and phosphate carrier protein (PCP) on the OMM, and the association of TSPO with the outer/inner mitochondrial membrane contact sites, drives the intramitochondrial cholesterol transfer and subsequent steroid formation [288].

## Conclusions

Steroid producing cells have a dual requirement for cholesterol: they need cholesterol for membrane biogenesis and cell signaling as well as starting material for the mitochondrial synthesis of pregnenolone, the precursor steroid required for the formation of glucocorticoids, mineralocorticoids, and sex-steroids. For steroid hormone production to proceed normally, adequate cholesterol must be available and supplied to the mitochondria. Under most physiological conditions, the supply of cholesterol is not rate-limiting, because there are multiple pathways that can fulfill the cholesterol needs of the cell. Although cellular *de novo *cholesterol synthesis and cholesteryl esters stored in lipid droplets can potentially supply adequate amounts of cholesterol substrate to support steroidogenesis, adrenal and ovary (and testicular Leydig cells under certain conditions), they however, preferentially utilize plasma lipoprotein-derived cholesterol for steroid synthesis. All steroidogenic cells irrespective of species have the capability to acquire cholesterol from plasma LDL, or other apolipoprotein B- (apoB) or apoE-containing lipoproteins via the well-characterized LDL (B/E) receptor-mediated endocytic pathway. Its functional efficiency, however, is dictated by the physiological status of the steroidogenic cell, the species and the type and composition of circulating lipoproteins. Steroidogenic cells can also process exceptionally large quantities of lipoprotein-derived cholesteryl esters through the "selective" cholesteryl ester uptake pathway. Indeed, the "selective" cholesterol uptake pathway is quantitatively the most important source for cholesterol delivery for steroidogenesis in the tropic-hormone stimulated rodent adrenal and ovary. The "selective" cholesterol uptake pathway involves internalization of cholesteryl esters from cell surface bound cholesterol-rich lipoproteins (HDL or LDL, regardless of lipoprotein composition) without the parallel uptake and lysosomal degradation of the lipoprotein particle itself. Hormone regulatable scavenger receptor class B, type I (SR-BI) is a physiologically relevant cell surface receptor responsible for "selective" uptake of lipoprotein-derived cholesteryl esters. The mechanisms by which plasma-lipoprotein cholesterol is delivered to steroidogenic cells via the SR-BI mediated "selective" uptake pathway has been extensively studied, but remains incompletely understood. Based on the current evidence, it appears that selective transfer of cholesterol esters to plasma membrane and their subsequent delivery to the cell interior by SR-BI requires the participation of accessory proteins, alterations in physicochemical characteristics of the plasma membrane (e.g., microvillar channel formation, caveolae/membrane lipid raft, lipid domain) and the specific physical form of SR-BI itself (dimerization, oligomerization).

The second step in cholesterol utilization for steroid hormone synthesis is intracellular cholesterol mobilization and processing and transport to the appropriate site within the mitochondria for side-chain cleavage and pregnenolone production. This process can be broadly divided into three separate, but equally important segments: a) tropic hormone-induced mobilization of cholesterol from intracellular stores, particularly from lipid droplets, transport of newly released free cholesterol to the outer mitochondrial membrane; and b) transfer of this cholesterol from the outer to the inner mitochondrial membrane for steroid (pregnenolone) production. Tropic hormone-mediated increased formation of the second messenger, cAMP, stimulates cAMP-PKA resulting in activation of cholesteryl ester hydrolase, and rapid hydrolysis of cholesteryl esters. The newly released cholesterol is transported to the outer mitochondrial membrane, although the actual underlying mechanism is not defined. Based on the currently available information, it appears that transport of hydrophobic cholesterol from the aqueous environment to OMM is primarily facilitated by the non-vesicular cholesterol transport mechanism involving StarD proteins such as the StarD4 and StarD5 family, which avidly bind cholesterol. In addition, cytoskeletal components/structures, particularly vimentin intermediate filaments, and direct interaction of lipid droplets to cellular organelles (e.g., mitochondria) and other cytosolic factors, steroidogenesis activator polypeptide (SAP) and changes in cellular architecture could also contribute to cholesterol transport to OMM.

The next step in cholesterol transport to mitochondria is the transfer from the outer to the inner mitochondrial membrane; this is considered as a rate-limiting step in hormone-induced steroid formation. Two proteins, translocator protein (18 kDa, TSPO) and steroidogenic acute regulatory (StAR) protein, which presumably work in concert, mediate this transfer. TSPO, previously known as the peripheral-type benzodiazepine receptor, is a high-affinity drug- and cholesterol-binding mitochondrial protein. StAR is a hormone-induced mitochondria-targeted protein that has been shown to initiate cholesterol transfer into mitochondria. The current thinking is that functional cooperation between TSPO and proteins such as the cAMP-dependent protein kinase regulatory subunitα (PKA-RIα) and the PKA-RIα- and TSPO-associated acyl-coenzyme A binding domain containing 3 (ACBD3) protein, PAP7, cholesterol is transferred to and docked at the OMM. The TSPO-dependent import of StAR into mitochondria, StAR interaction with VDAC1, and phosphate carrier protein (PCP) on the OMM, and the association of TSPO with the outer/inner mitochondrial membrane contact sites, drives the intramitochondrial cholesterol transfer and subsequent steroid formation.

## List of abbreviations used

**ACAT1**: acyl-coenzyme A:cholesterol acyltransferase 1; **ACTH**: adrenocorticotropin hormone; AII: angiotensin II; ANC: adenine nucleotide transporter; **AALO**: allopregnanolone; **CEH**: neutral cholesteryl ester hyrplase; **CE**: cholesteryl ester; **CEs**: cholesteryl esters; **CLA-1**: CD36 and LIMP-II analogues-1; **CLMP**: c-terminal linking and modulating protein; **CLP36**: *C*-terminal LIM domain protein 36; **CRAC**: domain; cholesterol recognition amino acid consensus domain; **DHEA**: dihydroepiandrosterone; **DHT**: dihydrotestosterone; **DHEA**: dehydroepiandrosterone; **Dlg**: the *Drosophila *discs large protein; **ECD**: extracellular domain; **E3KARP**: sodium hydrogen exchanger type 3 kinase A regulatory protein; **EBP50**: ERM binding protein 50; **ER**: endoplasmic reticulum; **ERM**: ezrin, the radixin and the moesin; **FERM**: Four-point one, ezrin, radixin and moesin; **FRET**: quantitative fluorescence resonance energy transfer; **FSH**: follicle-stimulating hormone; **hCG**: human chorionic gonadotropin; **HDL**: high-density lipoprotein; **HSL**: hormone-sensitive lipase; IF: vimentin-intermediate filaments; **IMM**: inner mitochondrial membrane; **LDL**: low-density lipoprotein; **LH**: Leuteinizing hormone; **NHERF1**: Na+/H+ exchange regulatory factor; **NHERF2**: Na+/H+ exchange regulatory factor 2; **NPC1**: Nieman-Pick type C1; **NPC2**: Nieman-Pick type C2; **OMM**: outer mitochondrial membrane; **NSF**: *N*-ethylmaleimide-sensitive factor; **OMM**: outer mitochondrial membrane; **ORPs**: OSBP-related proteins; **OSBP**: oxysterol-binding protein; **CYP11A1**: P450c11A; **CYP11B1**: P450c11; **CYP11B2**: aldosterone synthase; **CYP17**: P450c17; **CYP21A2**: P450c21; **PBR**: peripheral-type benzodiazepine receptor; **PDZ**: PSD-95, DglA, ZO-1; **PSD-95**: a 95 kDa protein involved in signaling in the post-synaptic density; **PAP7**: PKA regulatory subunit RIα-associated protein; **PKA**: cAMP-dpendent protein kinase; **POC**: proopiomelanocortin; **RGS12**: regulator of G protein signaling 12; **PRAX-1**: TSPO-associated protein-1; **RIL**: reversion-induced LIM protein; **SCAP**: SREBP cleavage-activating protein; **SCP_2_**: sterol career protein_2_; **α-SNAP**: soluble NSF attachment protein; **SNAP23**: synaptosomal-associated protein of 23; **SNAP25**: synaptosomal-associated protein of 25; **SNAREs**: SNAP receptors; **SR-BI**: Scavenger receptor Class B, type I; **SREBP**: sterol-regulatory element-binding proteins; **StAR**: steroidogenic acute regulatory protein; **START domain**: StAR-related lipid transfer domain; **THDOC**: allotetrahydrodeoxycortico; **TSPO**: translocator protein; **VAMP4**: vesicle-associated membrane protein4; **VDAC**: voltage-dependent anion channel; **ZO1**: the zonula occludens 1 protein involved in maintaining epithelial cell polarity;

## Competing interests

The authors declare that they have no competing interests.

## Authors' contributions

JH, ZZ, W-JS and SA provided background material and unpublished data for this review. SA drafted the manuscript. JH and ZZ contributed equally to this review. All authors read and approved the final manuscript.
